# Primaquine Pharmacokinetics in Lactating Women and Breastfed Infant Exposures

**DOI:** 10.1093/cid/ciy235

**Published:** 2018-03-24

**Authors:** Mary Ellen Gilder, Warunee Hanpithakphong, Richard M Hoglund, Joel Tarning, Htun Htun Win, Naw Hilda, Cindy S Chu, Germana Bancone, Verena I Carrara, Pratap Singhasivanon, Nicholas J White, François Nosten, Rose McGready

**Affiliations:** 1 Shoklo Malaria Research Unit, Mahidol-Oxford Tropical Medicine Research Unit, Mahidol University, Mae Sot; 2 Mahidol-Oxford Tropical Medicine Research Unit, Faculty of Tropical Medicine, Mahidol University, Bangkok, Thailand; 3 Centre for Tropical Medicine and Global Health, Nuffield Department of Medicine, University of Oxford, United Kingdom; 4 Department of Tropical Hygiene, Faculty of Tropical Medicine, Mahidol University, Bangkok, Thailand

**Keywords:** primaquine, lactation, breastfeeding, pharmacokinetic, malaria

## Abstract

**Background:**

Primaquine is the only drug providing radical cure of *Plasmodium vivax* malaria. It is not recommended for breastfeeding women as it causes hemolysis in glucose-6-phosphate dehydrogenase (G6PD)–deficient individuals, and breast milk excretion and thus infant exposure are not known.

**Methods:**

Healthy G6PD-normal breastfeeding women with previous *P. vivax* infection and their healthy G6PD-normal infants between 28 days and 2 years old were enrolled. Mothers took primaquine 0.5 mg/kg/day for 14 days. Primaquine and carboxyprimaquine concentrations were measured in maternal venous plasma, capillary plasma, and breast milk samples and infant capillary plasma samples taken on days 0, 3, 7, and 13.

**Results:**

In 20 mother–infant pairs, primaquine concentrations were below measurement thresholds in all but 1 infant capillary plasma sample (that contained primaquine 2.6 ng/mL), and carboxyprimaquine was likewise unmeasurable in the majority of infant samples (maximum value 25.8 ng/mL). The estimated primaquine dose received by infants, based on measured breast milk levels, was 2.98 µg/kg/day (ie, ~0.6% of a hypothetical infant daily dose of 0.5 mg/kg). There was no evidence of drug-related hemolysis in the infants. Maternal levels were comparable to levels in nonlactating patients, and adverse events in mothers were mild.

**Conclusions:**

The concentrations of primaquine in breast milk are very low and therefore very unlikely to cause adverse effects in the breastfeeding infant. Primaquine should not be withheld from mothers breastfeeding infants or young children. More information is needed in neonates.

**Clinical Trials Registration:**

NCT01780753.

## Teaser

Primaquine was excreted in very low levels in the breast milk of lactating women and caused no adverse effects in their infants. Restrictions on primaquine use in mothers breastfeeding infants at least 28 days old should be lifted.

**(See the Editorial Commentary by Price and Douglas on pages 1008–9.)**

Primaquine (PQ) is an 8-aminoquinoline used for the radical cure of *Plasmodium vivax* and *Plasmodium ovale* malaria, as a gametocytocide in falciparum malaria, and as malaria chemoprophylaxis [1, 2]. It is the only currently available drug that prevents *P. vivax* relapses. As malaria elimination becomes a global priority, support for the use of PQ has resurged [3].

*Plasmodium vivax* causes approximately 25% of malaria infections worldwide and its global economic cost in 2007 was estimated at 1–4 billion US dollars [4]. During pregnancy, infection with *P. vivax* contributes to maternal anemia and decreases birth weight [5, 6], which are associated with maternal [7] and infant mortality [8, 9]. Along the Thailand–Myanmar border, as in many areas of low unstable transmission outside sub-Saharan Africa, *P. vivax* is responsible for more than half of microscopy-proven malaria cases [10].

Treatment of *P. vivax* malaria is complicated by the presence of liver-stage parasites (hypnozoites) that may reactivate to cause illness weeks, months, or years after the initial illness. Hypnozoites cannot be treated by schizontocidal agents and, without radical cure by a 7- to 14-day course of PQ, infected individuals will often have multiple relapses. These relapses increase morbidity for affected individuals, burden the healthcare system, and contribute to ongoing transmission of *P. vivax* malaria, which hampers efforts to eliminate this parasite from endemic areas [11]. The most important adverse effect of PQ is hemolysis in glucose-6-phosphate dehydrogenase (G6PD)–deficient patients. The dose-dependent hemolytic anemia can be severe and life-threatening [12]. Other side effects include methemoglobinemia and abdominal discomfort.

There are limited data on the treatment of *P. vivax* infections in pregnant, postpartum, and breastfeeding women [13]. Since G6PD deficiency cannot be ruled out for the fetus in utero without invasive testing, PQ is contraindicated in pregnancy. Primaquine is well tolerated in G6PD-normal children 1 year and older [14], but data are limited for infants. There are no published data on the pharmacokinetics of PQ in lactating women, or on breast milk excretion. As a result, PQ is usually withheld from breastfeeding mothers to avoid the potential risk of iatrogenic hemolysis if the baby is G6PD deficient. Because of this uncertainty, the World Health Organization (WHO) 2015 malaria treatment guidelines recommend delaying radical cure for lactating women until their nursing infants are at least 6 months old and determined to be G6PD normal [1]. At the Thailand–Myanmar border, as in many malaria-endemic locations, extended breastfeeding [15] and G6PD deficiency [16] are common, and G6PD testing is often unavailable. As a result, many women are excluded for years from radical treatment as they continue breastfeeding. Frequently, a subsequent pregnancy begins before breastfeeding ends, eliminating any opportunity for radical cure and jeopardizing the next pregnancy.

More than 20 cases of hemolysis occurring in breastfed G6PD-deficient infants have been reported after maternal ingestion of prooxidant agents [17, 18]. While exposure through breast milk seems plausible in some cases, others involve agents that are not convincingly linked to hemolysis in G6PD-deficient individuals [19]. Importantly, reports of episodes occurring in the neonatal period do not account for the baseline incidence of unexplained hemolysis and severe jaundice in neonates with G6PD deficiency [20]. Evidence-based conclusions about the risks to breastfeeding infants should be based upon toxicokinetic studies.

This study was performed to quantify the infant’s drug exposure when a breastfeeding woman was treated with a 14-day course of PQ for the radical cure of *P. vivax* malaria.

## METHODS

### Study Setting and Population

This study took place at 3 of the Shoklo Malaria Research Unit (SMRU) clinics along the Thailand–Myanmar border, which serve migrant workers and refugees from Myanmar, predominantly from the Burman or Karen ethnic groups. Antenatal care and delivery services at SMRU clinics are described elsewhere [15]. Vivax malaria has been a common complication in pregnancy in this location and is associated with poor pregnancy outcomes [5, 6].

### Screening and Eligibility

Between 11 November 2012 and 24 June 2014, women ≥18 years old with a history of *P. vivax* infection and no prior radical cure PQ treatment who were breastfeeding healthy infants were invited to enroll in the study and receive a standard PQ radical cure regimen to prevent future relapses. Eligible mothers were counseled by trained staff following standard procedures for informed consent, accounting for preferred language and literacy, and women were allowed to give their decision at a subsequent visit, if needed. Primaquine administration was delayed until infants were at least 28 days old.

Complete blood count and G6PD testing were performed on consenting mothers and their babies. Both a rapid G6PD fluorescent spot test (R&D Diagnostic, Greece) and G6PD genotyping by polymerase chain reaction–restriction fragment length polymorphism for the most common local variant (Mahidol) were performed [16], and any abnormality in mother or baby led to exclusion. Maternal screening also included malaria smear, biochemistry, blood group, and hemoglobin typing.

### Ethical Considerations

This study was reviewed and approved by 3 ethical review bodies: the Tak Community Advisory Board [21]; the Ethics Committee of the Faculty of Tropical Medicine, Mahidol University in Bangkok (TMEC 12–036); and the Oxford Tropical Research Ethic Committee (OXTREC 28-12).

### Drug Administration

Primaquine GPO (Government Pharmaceutical Organization, Thailand) 0.5 mg base/kg was given to nonfasted women once daily for 14 days under directly observed therapy. Study staff saw participants daily while under treatment, recording any adverse events. In 1 case, staff went to the patient**’**s home to ensure daily dosing.

### Pharmacokinetic Sampling

Pharmacokinetic (PK) samples included maternal venous and capillary blood, breast milk, and infant capillary blood samples. Frequent PK samples were obtained on day 0 and day 13. On day 3 and day 7, sparse samples were taken (Supplementary Table 1). All blood samples were centrifuged on site and the plasma aliquots frozen immediately. Breast milk samples were obtained by manual expression. After measuring the total volume of expressed milk and mixing, a 2-mL aliquot was taken and frozen immediately. Samples were stored initially at –20°C and moved on dry ice to –80°C.

### Drug Level Measurement in Plasma and Breast Milk

Primaquine and carboxyprimaquine (CPQ) concentrations were quantified using a validated assay according to US Food and Drug Administration (FDA) guidelines (unpublished data). In brief, samples were processed by protein precipitation followed by phospholipid removal solid-phase extraction. Analytes were separated using reverse-phase high-performance liquid chromatography and quantified by electrospray ionization in the positive mode with multireaction monitoring mass spectrometry detection. The limits of quantification were 1.14 ng/mL for PQ and 4.88 ng/mL for CPQ. Three replicates of quality control samples at low, middle, and high concentrations were analyzed within each batch of clinical samples to ensure precision and accuracy during drug measurements. Total precision (ie, relative standard deviation) for all drug measurements was <10% during drug quantification of clinical samples.

### Pharmacokinetic Analysis

Individual drug concentrations on days 0 and 13 were analyzed using a noncompartmental analysis in Phoenix WinNonlin build 7.0.0.2535 (Certara, Princeton, New Jersey). The maximum concentration (C_max_) and the time to maximum concentration were derived from the observed PQ and CPQ concentrations. The observed data were used to calculate the exposure (area under the concentration–time curve [AUC]) from drug administration to the last observation and to infinity (AUC_∞_, only for PQ) based on the linear trapezoid method during absorption and logarithmic trapezoid method after C_max_. For PQ in venous plasma, oral elimination clearance and apparent oral volume of distribution were calculated based on the calculated AUC_∞_ and administered dose.

Hypothetical infant doses were calculated using 2 different approaches:

1. The dose was calculated based on breast milk PQ concentrations at the noted feeding and sampling occasion (measured in hours since PQ dose) multiplied by an estimated feeding volume of 33 mL/hour [22] since last noted concentration measurement.

$$Daily\;dose=\left(C_{T1}\times T_{1}\times 33\right)+\left(C_{T2}\times \left(T_{2}-\;T_{1}\right)\times 33\right)+\ldots$$ (1)

2. The total daily dose was calculated from:

$$Daily\;dose=\frac{AUC_{milk}}{AUC_{venous}}\times \frac{AUC_{venous\;\infty }}{Dosing\;interval}\times 150$$ (2)

One hundred fifty milliliters per kilogram per day is an estimate of infant breast milk consumption recommended as an industry standard [23]. Weight-adjusted relative infant dose was therefore:

$$Relative\;infant\;dose=\frac{Infant\;dose/kg}{Maternal\;dose/kg}\times 100$$ (3)

### Laboratory Monitoring

A Hawksley microhematocrit reader was used to measure hematocrit levels. Infant hematocrits were measured with each capillary PK sample, resulting in 2–4 hematocrit values on each sampling day. The mean of these 2–4 measurements for each infant on each day was used in the analysis. Heinz body counts and serum bilirubin measurements were also measured on each sampling day. Serum haptoglobin concentration on day 3 was measured by nephelometry. Methemoglobin was assessed using a transcutaneous probe (Masimo Radical-7). Heinz bodies per 1000 erythrocytes were assessed by microscopy after staining with 10% crystal violet. Serum bilirubin was measured using Bilimeter3 (Pfaff, Germany).

Infant study hematocrits were compared to longitudinal hematocrit data of 23 infants 7–10 weeks of age from this population.

### Statistical Analysis

Data were analyzed using Stata version 13.1 (StataCorp, College Station, Texas). Continuous normally distributed data were compared using the Student *t* test, and for nonnormally distributed data the Mann-Whitney *U* test was used. Wilcoxon signed-rank test compared paired nonparametric values (eg, day 0 vs day 13 hematocrit).

## RESULTS

Primaquine was well tolerated, and there were no obvious adverse effects in mothers or their infants.

### Participants

**Figure 1.**
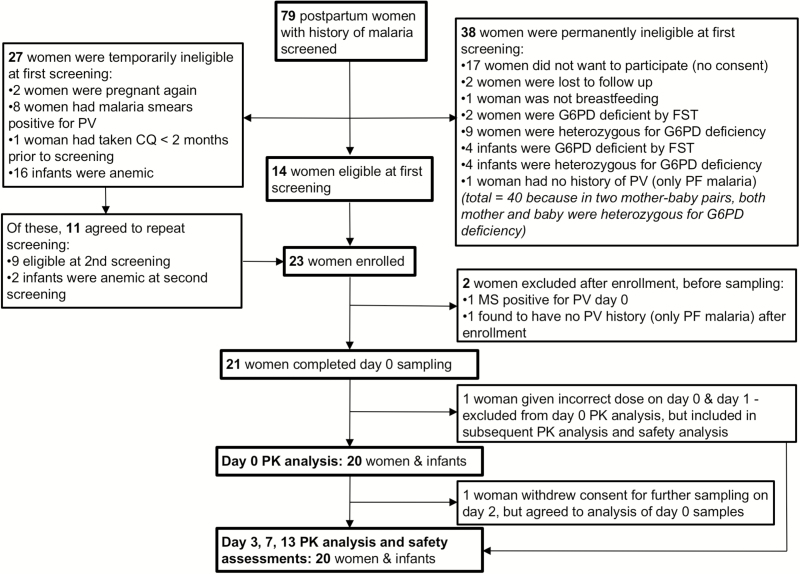
Inclusion flow diagram. Abbreviations: CQ, chloroquine; FST, fluorescent spot test; G6PD, glucose-6-phosphate dehydrogenase; MS, malaria smear; PF, *Plasmodium* falciparum; PK, pharmacokinetic; PV, *Plasmodium vivax.*

Twenty-one G6PD-normal mothers completed at least 1 day of pharmacokinetic sampling (Table 1), and 19 (90%) completed the full study according to the protocol (Figure 1; Supplementary Table 2). One woman withdrew consent for further sampling on day 2 because of pressure from family members to return home, but agreed to have previously collected (day 0) samples analyzed. She completed the course of PQ without complications. One participant was incorrectly given 1 mg/kg PQ for the first 2 days. She was excluded from day 0 PK analysis, but included for safety assessments (which were unremarkable) and PK analysis from day 3 onward.

**Table 1.** Baseline Demographic Characteristics (N = 21)

Characteristic		No. (%)
Mother’s age, y, median (range)		23 (18–40)
Ethnicity	Karen	16 (76)
	Burman	3 (14)
	Mixed	2 (10)
No. of children, median (range)		3 (1–9)
Duration of breastfeeding for previous children, mo, median (range)		18.5 (0–48)
Previous malaria episodes, median (range)		1 (1–4)
Proportion of patients with >1 episode		6/21 (29)
Age of infant, mo, median (range)		5 (1.5–22)
Infant sex, male		14 (67)
Proportion of infants <6 mo old introduced to water		12/13 (92)
Proportion of infants <6 mo old introduced to solid food		6/13 (46)
Proportion of infants >6 mo old introduced to both solid food and water		8/8 (100)

Data are presented as median (%) unless otherwise specified.

### Pharmacokinetic Data (Tables 2 and 3, Figures 2 and 3)

**Figure 2.**
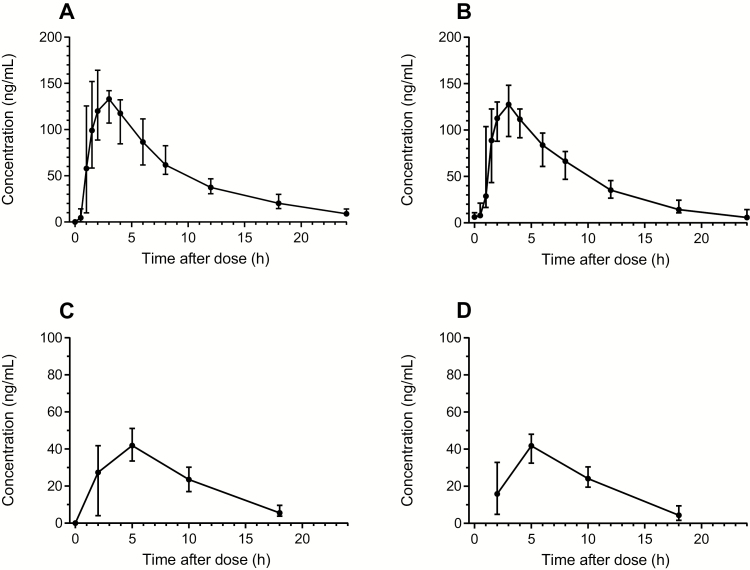
Concentration-time curves for primaquine. *A* and *B*, Venous primaquine samples. *C* and *D*, Primaquine concentrations in breast milk. Concentration-time curves have been stratified on sampling day 0 (*A* and *C*) and sampling day 13 (*B* and *D*). Mean and 95% confidence interval are shown.

**Figure 3.**
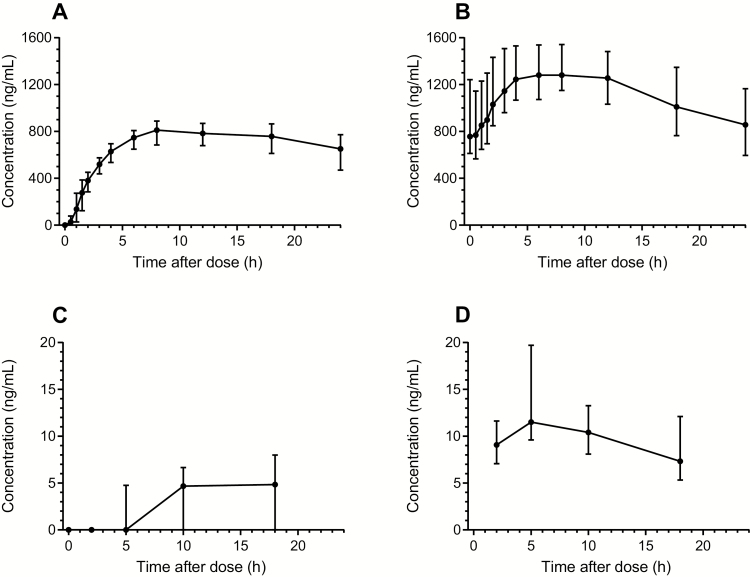
Concentration-time curves for carboxyprimaquine. *A* and *B*, Venous carboxyprimaquine samples. *C* and *D*, Carboxyprimaquine concentrations in breast milk. The concentration-time curves have been stratified on sampling day 0 (*A* and *C*) and sampling day 13 (*B* and *D*). Mean and 95% confidence interval are shown.

All infant capillary PQ concentrations were below the limit of quantification (LLOQ; 1.14 ng/mL or 2.28 ng/mL depending on the extracted volume) except for 1 sample that contained 2.59 ng/mL on day 7, hour 0. The PQ concentrations from the same baby at all other time points were below the LLOQ. Median CPQ concentrations were also below the LLOQ in infant samples (range, below the LLOQ to 25.8 ng/mL) (Table 2, Figures 2 and 3).

**Table 2.** Pharmacokinetic Parameters for Primaquine and Carboxyprimaquine in Venous Plasma and Breast Milk

Parameter	Primaquine		Carboxyprimaquine	
	Day 0	Day 13	Day 0	Day 13
Venous plasma				
T_max_, h	2.00 (0.983–4.02)	2.99 (1.05–4.00)	12.0 (6.00–18.00)	6.04 (3.00–17.6)
C_max_, ng/mL	139 (66.1–215)	132 (75.1–196)	833 (486–1280)	1370 (783–2250)
AUC_T_, h × ng/mL	1220 (499–2360)	1090 (460–2330)	16300 (9570–24800)	26900 (15300–45800)
AUC_∞_, h × ng/mL	1310 (529–3030)	1170 (475–2880)		
CL/F, L/h	23.5 (14.8–60.1)	25.8 (9.64–97.7)		
V/F, L	226 (102–458)	191 (117–625)		
Breast milk				
T_max_, h	3.42 (1.03–7.90)	3.87 (1.03–6.83)	19.4 (4.72–23.7)	4.17 (1.02–23.6)
C_max_, ng/mL	44.0 (31.4–99.0)	43.9 (26.8–116)	7.15 (3.98–19.4)	12.1 (7.02–28.4)
AUC_T_, h × ng/mL	420 (179–1150)	376 (164–1170)	100 (6.17–236)	159 (52.2–480)

Data are presented as median (range).

Abbreviations: AUC_∞_, area under the curve extrapolated to infinity; AUC_T_, area under the curve until last observation; CL/F, oral elimination clearance; C_max_, maximum concentration; T_max_, time to maximum concentration; V/F, apparent oral volume of distribution.

**Table 3.** Primaquine Pharmacokinetic Parameters Specific to Infant Feeding

Parameter	Day 0	Day 13
Maternal dose, mg/kg	0.50	0.50
Total daily infant dose, µg/kg^a^	1.62 (0.46–3.63)	1.17 (0.08–3.42)
Total daily infant dose, µg/kg^b^	2.98 (1.15–9.10)	2.58 (1.06–8.22)
Milk-plasma ratio, AUC_milk_/AUC_ven_	0.34 (0.12–0.64)	0.37 (0.24–0.61)
Relative infant dose, %^c^	0.618 (0.231–1.82)	0.517 (0.212–1.64)

Data are presented as median (range).

Abbreviations: AUC_milk_, area under the time-concentration curve until the last observation for breastmilk; AUC_ven_, area under the time-concentration curve until the last observation for venous plasma.

^a^Total daily dose per kilogram body weight of the baby, based on Equation 1 in Methods.

^b^Total daily dose per kilogram body weight of the baby, based on Equation 2 in Methods.

^c^Calculated based on Equations 2 and 3 in Methods.

Peak breast milk PQ concentrations were reached approximately an hour after peak plasma concentrations. The median total cumulative PQ dose expected to be ingested by the infant over the 14-day course based on measured breast milk concentrations was approximately 0.042 mg/kg, corresponding to 2.98 µg/kg/day (0.6% of a hypothetical infant daily dose of 0.5 mg/kg). The highest cumulative infant dose in this cohort was estimated at 0.127 mg/kg, or 9.07 µg/kg/day (1.8% of a hypothetical infant daily dose of 0.5 mg/kg) (Table 3).

### Safety Monitoring and Adverse Events in Mothers

The mothers’ median (range) baseline hematocrit was 38.5% (32%–43%). This decreased to 37% (30%–39%) by day 14 (*P* = .007), with a mean absolute change per week of –1.2% (95% confidence interval [CI], –2.1% to –.29%), corresponding to a mean fractional change per week of –2.9% (95% CI, –5.3% to –.48%) (Supplementary Figure 1*A*). Levels recovered to baseline values by day 21 without hematinics. Median methemoglobin (metHb) levels rose during the study period from 0.8% (range, 0.3%–1.5%) at baseline to 7.1% (range, 1.6%–11.9%) (*P* < .001), peaking on day 13. Between day 0 and day 14, at least 1 metHb value exceeded the normal limit (<3%) in 18 of 20 (90%) women, and 2 women had metHb levels >10% (maximum 12.6%). They experienced mild headache and fatigue, but symptoms resolved spontaneously and they completed their treatment.

### Safety Monitoring and Adverse Events in Infants

All infant hematocrits were within local population norms, and there was no symptomatic or severe anemia in the study infants. To assess change in hematocrit in infants of different ages over the period of possible PQ exposure, groups were formed based on age at enrollment: >56 days (n = 18) or ≤56 days (n = 2). This was done to account for the physiologic decline in hematocrit during the first months of life, which reaches its nadir typically between 8 and 12 weeks [24, 25] (Supplementary Figure 1*B*). Among infants >56 days old at enrollment, there was no change in hematocrit over the study period, with pretreatment median of 34.8% (range, 32.7%–38.7%) vs 34.3% (range, 32.0%–37.3%) on day 13 (*P* = .107).

The 2 infants enrolled at ≤56 days old had expected physiologic decreases in their hematocrit over the course of the study (Supplementary Table 3). The first infant, recruited at age 54 days, had a baseline hematocrit of 33.3% and a day 13 hematocrit of 30.3%, corresponding to a fractional change per week of –4.6%. The second infant, recruited at age 48 days, had a baseline hematocrit of 35.3% and a day 13 hematocrit of 32.3%, corresponding to a fractional change per week of –4.4%. This rate of fall was compared to fractional hematocrit change per week of 23 paired hematocrits from prospectively followed infants from the local population aged 7–10 weeks, and was within the interquartile ranges of this healthy cohort (mean, –2.8% [95% CI, –5.5% to .0]; interquartile range, –8.1% to 1.4%).

Haptoglobin levels were available for 14 of 20 infants (6 samples were rejected because of insufficient volume). Normal range for haptoglobin in infants is not well described: usually undetectable at birth, adult values of 0.3–2.0 g/L are reached by 4–6 months [26]. The haptoglobin of 1 infant enrolled at 48 days old was 0.1 g/L. Values in the other 13 infants measured fell in the normal adult range.

There was no correlation in either age group between PQ concentration in the breast milk and hematocrit change. Infant oxygen saturations, methemoglobin, serum bilirubin, and Heinz body counts also showed no significant changes over the study period. One infant >56 days old at enrollment had an asymptomatic abnormal metHb value of 4.4% on day 7, which resolved spontaneously while the mother took the remaining week’s treatment.

No other relevant adverse events or breastfeeding problems were reported.

## DISCUSSION

Most malaria treatment guidelines recommend that PQ be withheld from breastfeeding mothers lest their infants develop iatrogenic hemolytic anemia, although the amount of PQ excreted in breast milk has not been quantified previously. The unmeasurably low levels of PQ in all but 1 infant plasma sample in this study and negligible metabolite (CPQ) concentrations are very reassuring as they indicate very low exposure to maternal PQ. The very low breast milk PQ levels provided an estimated total weight-adjusted dose to the infant of <1% of the recommended adult and pediatric therapeutic dose. There is no evidence that PQ is more toxic to infants than adults (though information on the safety of PQ in infants is limited). Recent studies have shown that single PQ doses of 0.25 mg/kg do not cause clinically significant hemolysis in G6PD-deficient individuals [27]. It is highly improbable therefore that a total dose 6 times lower (0.042 mg/kg) spread over 14 days would cause hemolysis, even in G6PD-deficient infants.

The PK properties of PQ in these breastfeeding mothers were similar to those reported in nonpregnant adults [28–34], suggesting that dosing does not need adjustment during lactation.

None of the women screened for inclusion in this study had received previous radical cure. As a result, recurrent vivax malaria infection was common. This highlights the unmet need for PQ in breastfeeding women. Furthermore, 2 screened women, breastfeeding a 7-month-old and an 11-month-old, were excluded because of positive pregnancy tests, reflecting short interpregnancy intervals and repeat pregnancy before the end of lactation. Even with ideal interpregnancy intervals of 18–24 months [35], women following WHO recommendations to breastfeed for the first 2 years of life [36] would be ineligible for PQ throughout their reproductive life if treatment is withheld during lactation due to unknown or abnormal infant G6PD status.

There were no differences in absolute hematocrit or rate of change of hematocrit over time between infants enrolled in this study and age-matched infants from the local population. Lack of evidence of PQ toxicity was supported by evaluation of Heinz bodies, serum bilirubin, haptoglobin, and methemoglobin.

A limitation of this study is the approximate nature of breast milk volume estimation. Although FDA documents recommend collection of all breast milk from both breasts throughout the study period [23], this is burdensome for study participants, and pumped volumes of breast milk do not correlate well with intake by breastfeeding infants [22]. We asked women to empty 1 breast manually while feeding their infant on the other during preset sampling windows, and used published norms to calculate breast milk consumption. Expressed volumes varied from 5 mL to 101 mL, and differences in fat, protein, and oligosaccharide composition are inevitable. Analysis of foremilk vs hindmilk, or creamatocrit could give more detailed information. However, as the drug levels in all breast milk samples were low, and there was no association between drug concentration and volume of breast milk expressed or age of infant, these additional variables are unlikely to have clinical significance.

Further studies should test the predicted safety for G6PD-deficient infants suggested here by monitoring breastfed infants with G6PD deficiency during maternal PQ treatment. These reassuring results cannot be extrapolated to the neonatal period as breast milk during the first days after delivery differs significantly from mature milk. Studies of PQ treatment and colostrum PQ concentrations immediately postpartum, when many women have contact with healthcare providers, would be valuable.

New guidelines should allow PQ use in women breastfeeding infants at least 28 days old, and radical cure should be a routine part of 4- to 8-week postpartum care for women with a history of *P. vivax* malaria. This would reduce the risk of recurrent malaria in subsequent pregnancies, reduce morbidity and mortality due to malaria-related anemia, and help curb malaria transmission globally.

## Supplementary Data

Supplementary materials are available at *Clinical Infectious Diseases* online. Consisting of data provided by the authors to benefit the reader, the posted materials are not copyedited and are the sole responsibility of the authors, so questions or comments should be addressed to the corresponding author.

Supplementary Tables

Supplemental Figure 1

Supplementary Figure Legend

## Notes

***Author contributions.*** N. J. W., F. N., and R. M. conceptualized the study. W. H. and J. T. developed the assay to measure primaquine and carboxyprimaquine in breast milk and plasma. M. E. G., N. J. W., F. N., J. T., and R. M. developed the protocol. M. E. G., W. H., H. H. W., N. H., C. S. C., G. B., and V. I. C. generated the data. M. E. G., R. M. H., W. H., and J. T. analyzed the data and drafted the original manuscript. G. B., C. S. C., V. I. C., N. J. W., F. N., and R. M. made significant editorial contributions. All authors reviewed the final draft.

***Acknowledgments.*** The supportive collaboration and mentorship of Niklas Lindegardh throughout the early stages of this project were indispensable, and his memory continues to serve as an inspiration to the authors. This work is dedicated to his memory. The authors thank, above all, the women who agreed to participate in this study and the infants who were enrolled. We are grateful to all of the local staff working at the clinic level who identified and counseled eligible patients, provided patient care, and obtained samples and study data. We thank the laboratory staff and researchers in Mae Sot and Bangkok who processed and analyzed the samples, and experts from around the world who advised us. Julie Simpson and Ruth Lawrence both contributed their valuable expertise (in statistics and breastfeeding medicine, respectively) during the development of the protocol. Without this collaboration between village volunteers and academics in state-of-the-art universities and laboratories, this kind of research would not be possible.

***Disclaimer.*** The funding sources did not play a role in study design; collection, analysis, or interpretation of data; writing of the report; or the decision to submit the manuscript for publication.

***Financial support.*** Shoklo Malaria Research Unit is part of the Mahidol Oxford University Research Unit, supported by the Wellcome Trust of Great Britain. M. E. G. was supported by the National Institutes of Health Office of the Director, Fogarty International Center, Office of AIDS Research, National Cancer Center, National Eye Institute, National Heart, Blood, and Lung Institute, National Institute of Dental and Craniofacial Research, National Institute on Drug Abuse, National Institute of Mental Health, National Institute of Allergy and Infectious Diseases, and the Office of Women’s Health and Research through the Fogarty International Clinical Research Scholars and Fellows Program at Vanderbilt University (award number R24 TW007988) and the American Relief and Recovery Act. J. T. and R. M. H. were supported by the Bill & Melinda Gates Foundation (award number OPP1134284).

***Potential conflicts of interest.*** All authors: No reported conflicts of interest. All authors have submitted the ICMJE Form for Disclosure of Potential Conflicts of Interest. Conflicts that the editors consider relevant to the content of the manuscript have been disclosed.
