# Supplementary material for: Primaquine Pharmacokinetics in Lactating Women and Breastfed Infant Exposures
Source: Clin Infect Dis. 2018 Mar 24;67(7):1000–7. doi: 10.1093/cid/ciy235 (PMC6137118; doi:10.1093/cid/ciy235)
Supplement: Supplementary Tables [file ciy235_suppl_supplementary_tables.docx]

Supplementary Table 1. Pharmacokinetic (PK) sampling schedule

| Day | Time (hr) | Mother venous PK | Breast milk | Infant capillary PK |
| --- | --- | --- | --- | --- |
| 0 and 13 | 0 | X |  | X |
|  | 0.5 | X |  |  |
|  | 1 | X | 1-3 hrs after dose |  |
|  | 1.5 | X |  |  |
|  | 2 | X |  | X^a^ |
|  | 3 | X | 3-7 hrs after dose |  |
|  | 4 | X |  |  |
|  | 6 | X |  | X^a^ |
|  | 7 |  | 7-12 hrs after dose |  |
|  | 8 | X |  |  |
|  | 12 | X | 12-24 hrs after dose |  |
|  | 18 | X |  |  |
|  | 24 | X |  | X^ab^ |
| 3 and 7 | 0 | X | 1-3 hrs after dose | X |
|  | 2 | X |  | X^a^ |

^a^ Time for infant samples was measured from the time of the first feeding after maternal drug administration, e.g. if first breastfeeding occurred at maternal hr 1, hr 2 for the infant would correlate with maternal hr 3.

^b^ Infant sampling at hour 24 was done on day 13 only (not day 0)

**Supplementary Table 2.** Baseline hematologic indicators for safety analysis (n=20)

| Baseline maternal hematocrit (%) | 38.5 (32.0-43.0) |
| --- | --- |
| Baseline maternal methemoglobin (%) | 0.8 (0.3-1.5) |
| Baseline infant hematocrit (%) | 34.8 (32.7-38.7) |
| Baseline infant methemoglobin (%) | 0.5 (0-1.8) |
| Baseline infant Heinz bodies (per 1000 erythrocytes) | 0 (0-6) |
| Baseline infant serum bilirubin (umol/L) | 14 (0-59) |

Note: Data are presented as median (range) unless otherwise specified.

**Supplementary Table 3.** Results of infant hematologic monitoring during treatment

| Infant Hematologic markers | All Infants  (n=20) | compared with baseline | <56 days old (n=2)^a^ | compared with baseline^b^ | ≥56 days old (n=18) | compared with baseline |
| --- | --- | --- | --- | --- | --- | --- |
| Paired analysis of day 0 vs day 13 | | | | | | |
| HCT at day 13 (%) | 34.0  (30.3-37.3) | p=0.042 | 31.3  (30.3, 32.3) | p=0.157 | 34.3  (32.0-37.3) | p=0.107 |
| Median count Heinz bodies at day 13  (per 1,000 RBC) | 0 (0-7) | p=0.724 | 2  (2 for both) | p=0.317 | 0 (0-7) | p=0.979 |
| Median methemoglobin (%) at day 13 | 0.4 (0-2.8) | p=0.723 | 0.5 (0, 1.0) | p=0.655 | 0.4 (0-2.8) | p=0.632 |
| Serum bilirubin day 13 (umol/L) | 17 (6-33) | p=0.936 | 28 (26, 30) | p=0.655 | 16 (6-33) | p=0.831 |
| Single value on day 3 | | | | | | |
| Median haptoglobin (g/L) on day 3 | 0.8 (0.1-2.2) | N/A | 0.2 (0.1, 0.3) | N/A | 0.8 (0.3-2.2) | N/A |

Note: Data are presented as median (range) unless otherwise specified. Abbr: HCT Hematocrit, RBC red blood cell

^a^ n=2, exact values shown instead of ranges

^b^ significance of p value is limited by very small number of patients. See the patient level analysis of HCT in the text for more details.
