# Supplementary figures and images for: Primaquine Pharmacokinetics in Lactating Women and Breastfed Infant Exposures
Source: Clin Infect Dis. 2018 Mar 24;67(7):1000–7. doi: 10.1093/cid/ciy235 (PMC6137118; doi:10.1093/cid/ciy235)

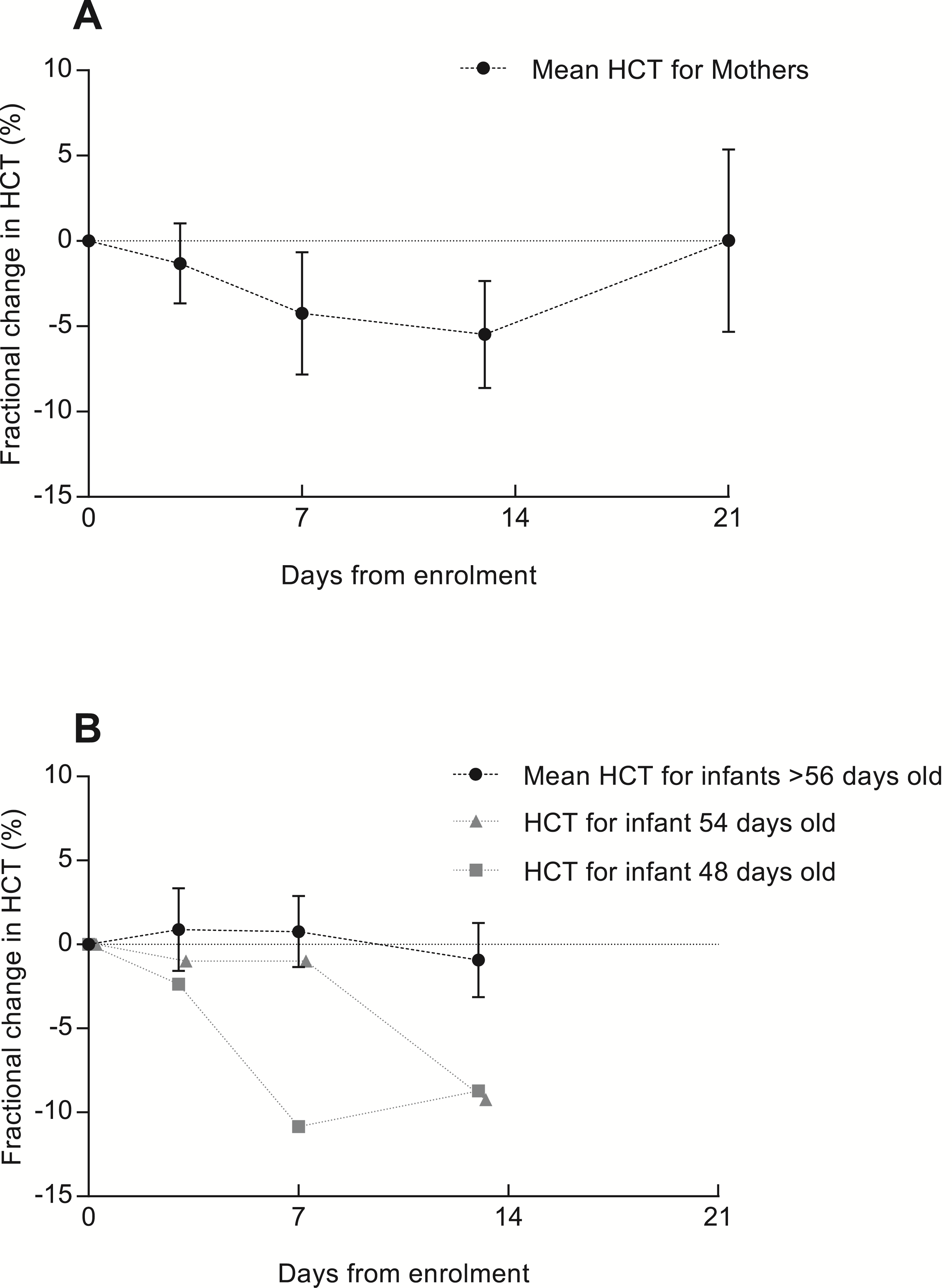

Supplement: Supplemental Figure 1 [file ciy235_suppl_supplemental_figure_1.png]
