# Supplementary material for: Primaquine Pharmacokinetics in Lactating Women and Breastfed Infant Exposures
Source: Clin Infect Dis. 2018 Mar 24;67(7):1000–7. doi: 10.1093/cid/ciy235 (PMC6137118; doi:10.1093/cid/ciy235)
Supplement: Supplementary Figure Legend [file ciy235_suppl_supplementary_figure_legend.docx]

**Supplementary Figure 1.** Maternal (A) and Infant (B) hematocrit changes during the study

Data on infants aged > 56 days (n=18) and mothers (n=20) are summarized with mean and 95% CI shown. The actual data of the infants < 56 days old (n=2) are plotted individually. Abbr: HCT Hematocrit.
